# Relationship of patients' age to histopathological features of breast tumours in *BRCA1 *and *BRCA2 *and mutation-negative breast cancer families

**DOI:** 10.1186/bcr1025

**Published:** 2005-04-21

**Authors:** Hannaleena Eerola, Päivi Heikkilä, Anitta Tamminen, Kristiina Aittomäki, Carl Blomqvist, Heli Nevanlinna

**Affiliations:** 1 Department of Oncology, Helsinki University Central Hospital, Finland; 2 Department of Obstetrics and Gynecology, Helsinki University Central Hospital, Finland; 3 Department of Pathology, Helsinki University Central Hospital, Finland; 4 Department of Clinical Genetics, Helsinki University Central Hospital, Finland

## Abstract

**Introduction:**

Our aim was to evaluate the relationship of patients' age to histopathological features of hereditary breast tumours in a series of breast cancer families not selected for age at diagnosis. In sporadic breast cancer, tumours from premenopausal patients have been shown to differ from those of postmenopausal patients, but this phenomenon has been little studied among familial patients.

**Methods:**

Representative areas of all available breast cancer tissue specimens (*n *= 262) from 25 *BRCA1*, 20 *BRCA2*, and 74 non-*BRCA1*/*2 *breast cancer families were punched into a tissue microarray. Immunohistochemical staining of oestrogen receptor, progesterone receptor, ERBB2, and p53 as well as the histology and grade of tumours in these three groups of families were studied in different age groups and compared with each other.

**Results:**

We found that only breast cancers from young (<50 years) *BRCA1*^+ ^patients represent features documented as being typical of *BRCA1*-associated cancers, such as high tumour grade, negativity for oestrogen and progesterone receptors, and overexpression of p53. Among the *BRCA2 *families, the opposite was found, with a significantly higher frequency of tumours negative for oestrogen and progesterone receptors among the older patients than among the other groups, but no distinctive tumour characteristics among the younger *BRCA2 *patients.

**Conclusion:**

Tumours of *BRCA1 *and *BRCA2 *carriers aged 50 years or more differed significantly from those of younger carriers. This difference may reflect different biological behaviour and pathways of tumour development among the older and the younger *BRCA1 *and *BRCA2 *patients, with impact also on prognosis and survival.

## Introduction

Distinct pathological features among *BRCA1*-associated tumours have been found when such tumours are compared with sporadic cancers; these features include high tumour grade, negativity for oestrogen receptor (ER), overexpression of p53, negativity for progesterone receptor (PR), and a higher proportion of medullary and atypical medullary carcinomas [1-3]. Recently, cDNA expression analyses have suggested a basal epithelial phenotype for *BRCA1 *tumors [4] and expression of cytokeratins 5/6 have been associated with *BRCA1 *tumours [5]. Among *BRCA2*-associated tumours, findings have been inconsistent, and in most cases no significant difference has been found between *BRCA2*-associated and sporadic cancers [1,2,6,7].

In our previous report [8], we have shown, consistent with earlier studies, that *BRCA1*-associated cancers were diagnosed younger and were more ER^- ^and progesterone-receptor-negative (PR^-^), more p53^+^, and of higher grade than unselected breast tumours or tumours from non-*BRCA1*/*2 *breast cancer families. However, in multivariate analysis the independent factors, as compared with non-*BRCA1*/*2 *tumours, were age of diagnosis, grade, and PR-negativity. *BRCA2 *cases did not have such distinctive features compared with non-*BRCA1*/*2 *cases.

In large studies on sporadic breast cancer tumours, tumours from premenopausal patients have been shown to differ from those of postmenopausal patients [9-11], but this has been little studied among familial patients. In this study, we had an excellent opportunity to study familial cases without age restriction and to evaluate whether the histology and immunohistochemistry differ in the different age groups (according to whether age of diagnosis is below or over 50 years, with age being used as a surrogate for menopause status) among *BRCA1*, *BRCA2*, and non-*BRCA1*/*2 *families.

## Materials and methods

Family history of cancer was screened for among breast cancer patients in the Department of Oncology, Helsinki University Central Hospital [12]. Families were collected with a simple criterion of at least three first- or second-degree relatives with breast or ovarian cancer, with no restriction regarding age. All the families were tested for *BRCA1 *and *BRCA2 *mutations by mutation analysis of the whole coding sequences and exon/intron boundaries of the genes as described elsewhere [13,14], or were tested for all 18 previously reported Finnish *BRCA1 *and *BRCA2 *mutations [13-16]. In this study, as previously described [8], we collected all the available paraffin-wax blocks of the primary breast cancers (*n *= 262) from 119 breast cancer families. Altogether, 51 cancers from the 25 *BRCA1 *families, 59 cancers from the 20 *BRCA2 *families, and 152 cancers from the 74 non-*BRCA1*/*2 *families were obtained.

The patients' median age at diagnosis of the tumours was 44 years for *BRCA1*, 47 years for *BRCA2*, and 55 years for non-*BRCA1*/*2*. For comparison of tumours from premenopausal and postmenopausal patients, the age of 50 years was chosen as a surrogate for menopause. Among the *BRCA1 *patients, 34 (66.7%) were diagnosed when they were below 50 years of age (median age 39) and 17 (33.3%) when they were 50 or more (median age 55); the respective numbers among *BRCA2 *patients were 35 (59.3%) and 24 (40.7%) (median ages 39 and 56.5, respectively), and among non-*BRCA1*/*2 *patients, 58 (38.2%) and 94 (61.8%) (median ages 44 and 65, respectively).

The most representative area of the tumour was punched to produce a hereditary breast cancer tissue microarray including two cores (diameter 0.6 mm) from all the original blocks as described elsewhere [8,17]. The use of microarray tissue blocks makes it possible to stain all the samples at the same time and in the same conditions. Subgroups of the material are therefore very well comparable, and a highly significant correlation between this kind of multicore system and studying the whole sections of the original blocks has also been shown [18,19].

All the tissue microarray slides were stained with routine methods used for pathological diagnostics with ER, PR, ERBB2, and p53 antibodies in the same laboratory [8]. Briefly, five-micrometer sections were cut from paraffin-embedded blocks, dewaxed in xylene, and dehydrated in a series of graded alcohols. The sections were pretreated in a microwave oven and incubated with antibody overnight. ER antibody (1:50) and ERBB2 antibody (NCL-CB11, 1:400) were purchased from Novocastra (Newcastle upon Tyne, UK), and PR (1:250) and p53 antibodies (1:100) were from Dako (Copenhagen, Denmark). The evaluation of the staining results was similar to that used in routine diagnostics, and samples were considered positive when 10%, 10%, and 20% of the cells were stained with ER, PR, and p53, respectively. Samples having a moderate or intense staining of the entire membrane in more than 10% of the tumour cells (immunohistochemical scores of 2+ and 3+) were considered to be ERBB2^+^. Other staining patterns (0 and 1+) were considered to be negative. We studied the haematoxylin-and-eosin sections of the original blocks to achieve histological diagnosis and grading (all by the same pathologist (PH)). Statistical analysis was done with SPSS version 8.0 for Windows. We tested the differences in dichotomous variables with a χ^2 ^or Fisher's exact test. All *P *values are two-tailed.

Permissions for this study were obtained from the ethics committees of the Department of Oncology and the Department of Obstetrics and Gynaecology, Helsinki University Central Hospital, and of the Ministry of Social Affairs and Health in Finland. Blood and tumour samples were used in this study with the informed consent of the probands and of the family members.

## Results

In *BRCA1 *families, patients whose cancer was diagnosed when they were under 50 years of age differed significantly from those diagnosed at 50 years or older in the presence of grade 3 tumours (84.4% vs 47.1%, *P *= 0.009), ER-negativity (83.3% vs 25%, *P *= 0,001), and p53-positivity (50.0% vs 7.7%, *P *= 0.014) (Table 1). All of the five cancers with medullary histology were also detected in patients under 50 years old. Patients who were *BRCA1*^+ ^and were under 50 years old at diagnosis differed significantly in all of these factors from familial non-*BRCA1*/*2 *patients (proportion of grade 3 tumours, 84.4% vs 17.3%, respectively, *P *≤ 0.0005; of ER-negativity, 83.3% vs 29.3%, *P *≤ 0.0005; of PR-negativity, 90.3% vs 31.0%, *P *≤ 0.0005; and of p53-positivity, 50.0% vs 25.9%, *P *= 0.024). However, patients from *BRCA1 *families diagnosed at age 50 years or older differed significantly only for grade from the non-*BRCA1*/*2 *patients in the same age group (47.1% vs 23.3, *P *= 0.044).

In *BRCA2 *families, tumours of patients diagnosed at less than 50 years of age differed significantly from those of the older patients for ER-negativity (20.6% vs 52.6%, respectively, *P *= 0.017) and PR-receptor negativity (35.3% vs 80.0%, *P *= 0.001) (Table 1). In contrast to *BRCA1 *tumours, the *BRCA2 *tumours diagnosed in patients 50 years or older were more often ER^- ^(52.6% vs 25.6%, *P *= 0.02) and PR^- ^(80.0% vs 54.4%, *P *= 0.036) than non-*BRCA1*/*2 *cancers among the same age group. Tumours of patients diagnosed at less than 50 years of age were very similar to non-*BRCA1*/*2 *tumours in the same age group (Table 1).

Pathological features of non-*BRCA1*/*2 *tumours did not vary significantly between the two age groups.

## Discussion

In this study, we have evaluated whether tumour histology and immunohistochemistry are influenced by age of onset (menopause status) among families with *BRCA1*, *BRCA2*, or non-*BRCA1*/*2 *tumours. Most of the earlier studies of the characteristics of tumours in *BRCA1 *and *BRCA2 *carriers have been based on young patients only. Because there was no age restriction in our selection criterion, we had an excellent opportunity to study patients within the whole age distribution.

In *BRCA1 *families, tumours from patients diagnosed at over 50 years of age were surprisingly different from those in *BRCA1 *carriers diagnosed at under 50 years. Only tumours from the younger patients exhibited the distinctive characteristics that have been found to be typical of *BRCA1 *tumours, with higher grade, negativity for ER and PR, and positivity for p53 distinguishing them from familial non-*BRCA1*/*2 *tumours. However, tumours from the older patients in *BRCA1 *families differed significantly only in grade from tumours in non-*BRCA1*/*2 *patients. There were only five cases among this older group of patients, for which the *BRCA1 *mutation status was unknown. If these patients are excluded from the analysis, the observed frequencies remain ; therefore those do not account for the result.

Previously, Vaziri and colleagues [20] have reported that the tumour immunophenotype of *BRCA1*-carriers is influenced by the age of diagnosis. As a control group, those authors used age-matched breast cancer patients unselected for family history, whereas in our study we included familial non-*BRCA1*/*2 *cancer cases. Vaziri and colleagues observed no differences in ER or PR staining of tumours between *BRCA1 *carriers diagnosed at 50 years or older and controls with sporadic cancers [20]. Foulkes and colleagues [21] also recently reported that the proportion of ER^+ ^tumours increased with patients' age among the *BRCA1 *patients included in their study (diagnosed at less than 65 years of age), although they found a strong relationship between *BRCA1 *carrier status and ER-negativity of tumours in the age group 55 to 65 years. Vaziri and colleagues also studied the expression of markers Ki-67, Cyclin D1, p53, and ERBB2. None of these markers differed significantly in the patients 50 years or older between *BRCA1*-associated cancers and control cancers, although tumours from the younger *BRCA1 *age group presented less frequent ER, PR, and cyclin D1 staining and more frequent Ki-67 and B-catenin staining than those from control cancers. p53 expression did not differ in their study in different age groups, nor was p53 more frequently overexpressed among young *BRCA1 *patients than in controls.

We did not find the *BRCA2*-associated tumours to differ significantly from familial non-*BRCA1*/*2 *tumours among the younger age group. However, tumours of *BRCA2 *carrier patients diagnosed at 50 years or older had more distinctive features, and were more ER^- ^and PR^-^, than tumours of younger patients or tumours of the same age group of *BRCA1 *patients or non-*BRCA1*/*2 *patients.

The specific features of *BRCA1*-associated tumours among the younger age group, and lack of such features among the *BRCA2*-associated tumours, are consistent with the overall characteristics reported previously among *BRCA1 *and *BRCA2 *patients [1,2]. Such features characterise to a large extent the *BRCA1 *and *BRCA2 *tumours overall, as a large majority (63% in this study) of all breast tumours in the *BRCA1 *and *BRCA2 *families are diagnosed before patients reach 50 years of age.

However, among both *BRCA1 *and *BRCA2 *families, tumours from older patients form subgroups that are distinctly different from those of the younger patients. Tumours from the older *BRCA1 *patients resemble more those among the mutation-negative families, or sporadic tumours. The highest incidence rates and relative breast cancer risk among *BRCA1 *carriers are seen before age 50 [22], and some tumours from older *BRCA1 *mutation carriers could also be 'sporadic' cancers. However, the breast tumours from older *BRCA1 *patients also differed from mutation-negative ones by their higher grade. Furthermore, tumours from the older *BRCA2 *carriers exhibited distinctly different characteristics from the younger ones or from *BRCA1 *carrier tumours and mutation-negative ones, suggesting a strong impact of the germline mutation on tumour development among the older patients. It is interesting that the *BRCA1 *and *BRCA2 *tumours appear to be opposites with respect to their characteristics in the age groups of younger and older patients.

There are now many models and computer programs to test the probability of *BRCA1 *or *BRCA2 *mutations [23-28]. We have also documented previously that efficient predictors for *BRCA1 *and *BRCA2 *mutations are early age of breast cancer onset and number of ovarian cancer cases in the family [27]. Simple family history criteria of the strongest predictors (onset of breast cancer under age 40 and presence of ovarian cancer) for a mutation may also provide a rough estimate of a high likelihood of carrying a mutation [27].

However, it would be useful if, besides family history, histopathological markers could also be used to distinguish patients and families likely to carry a *BRCA1*/*2 *germline mutation from mutation-negative families and breast cancer patients in general. The use of morphologic and immunohistochemical data has been previously suggested to provide a helpful and cost-effective tool for predicting *BRCA1 *mutation among young breast cancer patients [2,29,30]. The findings here provide further information specifically with respect to older *BRCA1 *and *BRCA2 *patients and warrant further studies for evaluating the probability of mutation by combining information on family history and tumour characteristics in the various age groups.

## Conclusion

These findings may reflect different biological behaviour and pathway of tumour development among the older and the younger *BRCA1 *and *BRCA2 *patients, with impact also on prognosis and survival. So far, results on survival among *BRCA1 *and *BRCA2 *patients have been inconclusive or contradictory, and large meta-analyses specifically according to age groups could shed further light on this. Finally, in the context of genetic counselling, specific tumour characteristics may help evaluate the possibility of a *BRCA1 *or *BRCA2 *mutation and the need for mutation testing in a family with a history of breast cancer. It appears crucial, however, to consider such features specifically with respect to the age of the patients.

## Abbreviations

ER = oestrogen receptor; PR = progesterone receptor.

## Competing interests

The author(s) declare that they have no competing interests.

## Authors' contributions

HE drafted the manuscript, participated in the design of the study and data collection, and performed the statistical analysis. PH carried out the immunohistochemistry. AT carried out the molecular genetic studies. KA participated in patient collection and did the genetic counselling of the patients and participated in drafting the manuscript. CB participated in the design of the study and drafting of the manuscript. HN participated in the design of the study, data collection, and drafting of the manuscript. All authors read and approved the final manuscript.

## Figures and Tables

**Table 1:** Features of breast tumours according to breast cancer patient's age (years) at diagnosis

	*BRCA1 *tumours					*BRCA2 *tumours					Non-*BRCA1/2 *tumours				
	
	Age <50		Age ≥ 50		*P *< 0.05^a^	Age <50		Age ≥ 50		*P *< 0.05^a^	Age <50		Age ≥ 50		*P *< 0.05^a^
Histology															
Ductal cancer	24	(70.6)	13	(76.5)		19	(54.3)	18	(75.0)		34	(58.6)	68	(72.3)	
Lobular cancer	5	(14.7)	3	(17.6)		12	(34.3)	5	(20.8)		15	(25.9)	15	(16.0)	
Medullary cancer	5	(14.7)									2	(3.4)	1	(1.1)	
Other cancers			1	(5.9)		4	(11.4)	1	(4.2)		7	(12.1)	10	(10.6)	
Grade															
I	1	(3.1)	2	(11.8)		7	(23.3)	5	(22.7)		13	(25.0)	33	(36.7)	
II	4	(12.5)	7	(41.2)		16	(53.3)	10	(45.5)		30	(57.7)	36	(40.0)	
III	27	(84.4)	8	(47.1)	0.009	7	(23.3)	7	(31.8)		9	(17.3)	21	(23.3)	
I to II	5	(16.6)	9	(53.0)		13	(76.7)	15	(68.2)		43	(82.7)	69	(76.7)	
Immunohistochemistry															
ER^-^	25	(83.3)	3	(25.0)	0.001	7	(20.6)	10	(52.6)	0.017	17	(29.3)	23	(25.6)	
ER^+^	5	(16.7)	9	(75.0)		27	(79.4)	9	(47.4)		41	(70.7)	67	(74.7)	
PR^-^	28	(90.3)	9	(69.2)		12	(35.3)	16	(80.0)	0.001	18	(31.0)	49	(54.4)	
PR^+^	3	(9.7)	4	(30.8)		22	(64.7)	4	(20.0)		40	(69.0)	41	(45.6)	
p53^-^	15	(50.0)	12	(92.3)	0.014	27	(81.8)	15	(83.3)		43	(74.1)	75	(81.5)	
p53^+^	15	(50.0)	1	(7.7)		6	(18.2)	3	(16.7)		15	(25.9)	17	(18.5)	
ERBB2^-^	23	(76.7)	12	(92.3)		29	(83.3)	16	(84.2)		45	(81.8)	64	(83.1)	
ERBB2^+^	7	(23.3)	1	(7.7)		5	(14.7)	3	(15.8)		10	(18.2)	13	(16.9)	

Values are no. (frequency %). ^a^Comparison by age group. There were no significant differences according to patients' age in the non-*BRCA1/2 *tumours (rightmost column). ER, oestrogen receptor; ERBB2, *ERBB2 *oncoprotein; PR, progesterone receptor.
